# Supplementary material for: Change in five-factor model personality traits during the acute phase of the coronavirus pandemic
Source: PLoS One. 2020 Aug 6;15(8):e0237056. doi: 10.1371/journal.pone.0237056 (PMC7410194; doi:10.1371/journal.pone.0237056)
Supplement: S1 Fig — (DOCX) [file pone.0237056.s001.docx]

Figure S1

*Flow Chart of Participants Inclusion/Exclusion*

|  |  | Time 1 |  |  | Time 2 |  |
| --- | --- | --- | --- | --- | --- | --- |
| Responses to survey |  | 5186 |  |  | 2538 |  |
| Consent: No | 467 |  |  | 40 |  |  |
|  |  | 4719 |  |  | 2498 |  |
| Duplicate ID entries | 55 |  |  | 9 |  |  |
|  |  | 4664 |  |  | 2489 |  |
| Missing personality responses | 491 |  |  | 147 |  |  |
|  |  |  |  |  | 2342 |  |
| Drop-out of panel/Invited for time 2 | 324 |  | 3815 |  |  |  |
|  |  |  |  |  |  |  |
| Demographic inconsistencies | 60 |  |  | 60 |  |  |
| Repetitive responses/>20% missing | 154 |  |  | 94 |  |  |
| Sample at each time |  | 3959 |  |  | 2189 |  |
| Analytic longitudinal sample |  |  | 2137 |  |  |  |
