# Supplementary material for: Change in five-factor model personality traits during the acute phase of the coronavirus pandemic
Source: PLoS One. 2020 Aug 6;15(8):e0237056. doi: 10.1371/journal.pone.0237056 (PMC7410194; doi:10.1371/journal.pone.0237056)
Supplement: S1 Table — a Ns vary due to missing data. ^ From Roberts et al. [14]. (DOCX) [file pone.0237056.s002.docx]

Table S1

*Mean Change in Personality Traits between Pretest and Posttest Controlling for Covariates*

| Personality Trait |  | Pretest | | Posttest | | Time | *p* | *η*^2^ |
| --- | --- | --- | --- | --- | --- | --- | --- | --- |
|  |  | Mean | SE | Mean | SE |  |  |  |
| Neuroticism |  | 2.554 | .053 | 2.484 | .054 | *F*(1,2127)=5.106 | .024 | .003 |
| Extraversion |  | 3.199 | .046 | 3.176 | .046 | *F*(1,2127)=.103 | .748 | .000 |
| Openness |  | 3.492 | .045 | 3.513 | .046 | *F*(1, 2127)=.684 | .408 | .001 |
| Agreeableness |  | 3.735 | .043 | 3.722 | .044 | *F*(1, 2127)=2.568 | .109 | .001 |
| Conscientiousness |  | 3.961 | .048 | 3.936 | .049 | *F*(1, 2127)=1.399 | .237 | .000 |
| Neuroticism Facets |  |  |  |  |  |  |  |  |
| Anxiety |  | 2.868 | .061 | 2.810 | .062 | F(1,2127)=6.910 | .009 | .003 |
| Depression |  | 2.349 | .061 | 2.303 | .062 | F(1,2127)=.741 | .389 | .001 |
| Emotional Volatility |  | 2.445 | .059 | 2.339 | .060 | F(1,2127)=2.331 | .127 | .001 |
| Extraversion Facets |  |  |  |  |  |  |  |  |
| Sociability |  | 2.963 | .065 | 2.873 | .065 | *F*(1,2127)=.111 | .739 | .000 |
| Assertiveness |  | 3.307 | .055 | 3.300 | .056 | *F*(1,2127)=.725 | .395 | .000 |
| Energy Level |  | 3.326 | .055 | 3.357 | .054 | *F*(1,2127)=.034 | .853 | .000 |
| Openness Facets |  |  |  |  |  |  |  |  |
| Curiosity |  | 3.562 | .052 | 3.574 | .052 | *F*(1,2127)=.423 | .515 | .000 |
| Aesthetic Sensitivity |  | 3.368 | .059 | 3.371 | .060 | *F*(1,2127)=.324 | .569 | .000 |
| Creative Imagination |  | 3.545 | .055 | 3.593 | .056 | *F*(1,2127)=.234 | .629 | .000 |
| Agreeableness Facets |  |  |  |  |  |  |  |  |
| Compassion |  | 3.813 | .052 | 3.791 | .053 | *F*(1,2127)=2.295 | .130 | .001 |
| Respectfulness |  | 4.058 | .052 | 4.013 | .052 | *F*(1,2127)=3.394 | .047 | .003 |
| Trust |  | 3.336 | .053 | 3.363 | .053 | *F*(1,2127)=.011 | .917 | .000 |
| Conscientiousness Facets |  |  |  |  |  |  |  |  |
| Organization |  | 3.984 | .058 | 3.942 | .058 | (1,2127)=4.698 | .030 | .001 |
| Productiveness |  | 3.938 | .056 | 3.925 | .056 | (1,2127)=.902 | .342 | .001 |
| Responsibility |  | 3.960 | .051 | 3.940 | .052 | (1,2127)=.345 | .557 | .000 |
| Responsibility^a^^ |  | 4.004 | .050 | 3.917 | .051 | (1,2055)=3.088 | .079 | .001 |
| Dutifulness^a^ |  | 3.877 | .042 | 3.831 | .042 | (1,2024)=.125 | .724 | .000 |

*Note*. *N*=2,137. ^a^ Ns vary due to missing data. ^ From Roberts et al., 2005.
