# Supplementary material for: Change in five-factor model personality traits during the acute phase of the coronavirus pandemic
Source: PLoS One. 2020 Aug 6;15(8):e0237056. doi: 10.1371/journal.pone.0237056 (PMC7410194; doi:10.1371/journal.pone.0237056)
Supplement: S2 Table — N = 2,137. a Ns vary due to missing data. ^ From Roberts et al. [14]. (DOCX) [file pone.0237056.s003.docx]

Table S2

*Interaction Between Time and Age on Change in Personality Traits*

| Personality Trait |  | Pre |  | Post |  | Time | Age | Time x Age |
| --- | --- | --- | --- | --- | --- | --- | --- | --- |
|  |  | Mean | SD | Mean | SD |  |  |  |
| Neuroticism |  |  |  |  |  |  |  |  |
| <65 |  | 2.729 | .788 | 2.683 | .801 | *F*(1,2135)=4.434, | *F*(1,2135)=150.871, | *F*(1,2135)=2.874, |
| ≥65 |  | 2.259 | .678 | 2.254 | .705 | *p*=.035 | *p*=.000 | *p*=.090 |
| Extraversion |  |  |  |  |  |  |  |  |
| <65 |  | 3.093 | .647 | 3.108 | .640 | *F*(1,2135)=5.214, | *F*(1,2135)=13.738, | *F*(1,2135)=.585, |
| ≥65 |  | 3.200 | .617 | 3.229 | .652 | *p*=.023 | *p*=.000 | *p*=.444 |
| Openness |  |  |  |  |  |  |  |  |
| <65 |  | 3.429 | .622 | 3.437 | .628 | *F*(1,2135)=2.004, | *F*(1,2135)=12.575, | *F*(1,2135)=.460, |
| ≥65 |  | 3.528 | .625 | 3.549 | .665 | *p*=.157 | *p*=.000 | *p*=.498 |
| Agreeableness |  |  |  |  |  |  |  |  |
| <65 |  | 3.609 | .628 | 3.605 | .641 | *F*(1,2135)=.009, | *F*(1,2135)=134.844, | *F*(1,2135)=.095, |
| ≥65 |  | 3.950 | .594 | 3.952 | .605 | *p*=.925 | *p*=.000 | *p*=.759 |
| Conscientiousness |  |  |  |  |  |  |  |  |
| <65 |  | 3.780 | .720 | 3.772 | .733 | *F*(1,2135)=.485, | *F*(1,2135)=100.599, | *F*(1,2135)=2.168, |
| ≥65 |  | 4.097 | .594 | 4.121 | .628 | *p*=.486 | *p*=.000 | *p*=.141 |
|  |  |  |  |  |  |  |  |  |
| Anxiety |  |  |  |  |  |  |  |  |
| <65 |  | 3.012 | .886 | 2.954 | .897 | *F*(1,2135)=5.639, | *F*(1,2135)=87.602, | *F*(1,2135)=1.343, |
| ≥65 |  | 2.610 | .851 | 2.590 | .853 | *p*=.018 | *p*=.000 | *p*=.247 |
| Depression |  |  |  |  |  |  |  |  |
| <65 |  | 2.559 | .905 | 2.518 | .923 | *F*(1,2135)=3.631, | *F*(1,2135)=151.194, | *F*(1,2135)=.542, |
| ≥65 |  | 2.041 | .761 | 2.023 | .791 | *p*=.057 | *p*=.000 | *p*=.442 |
| Emotional Volatility |  |  |  |  |  |  |  |  |
| <65 |  | 2.614 | .886 | 2.576 | .893 | *F*(1,2135)=.184, | *F*(1,2135)=134.637, | *F*(1,2135)=3.598, |
| ≥65 |  | 2.124 | .711 | 2.148 | .760 | *p*=.668 | *p*=.000 | *p*=.058 |
| Sociability |  |  |  |  |  |  |  |  |
| <65 |  | 2.885 | .899 | 2.869 | .896 | *F*(1,2135)=1.787, | *F*(1,2135)=13.874, | *F*(1,2135)=.049, |
| ≥65 |  | 3.047 | .893 | 3.024 | .910 | *p*=.181 | *p*=.000 | *p*=.825 |
| Assertiveness |  |  |  |  |  |  |  |  |
| <65 |  | 3.153 | .774 | 3.187 | .776 | *F*(1,2135)=7.580, | *F*(1,2135)=1.616, | *F*(1,2135)=.174, |
| ≥65 |  | 3.193 | .759 | 3.239 | .798 | *p*=.006 | *p*=.204 | *p*=.676 |
| Energy Level |  |  |  |  |  |  |  |  |
| <65 |  | 3.241 | .778 | 3.268 | .759 | *F*(1,2135)=9.305, | *F*(2,2135)=15.232, | *F*(1,2135)=1.560, |
| ≥65 |  | 3.358 | .708 | 3.423 | .755 | *p*=.002 | *p*=.000 | *p*=.212 |
| Curiosity |  |  |  |  |  |  |  |  |
| <65 |  | 3.520 | .729 | 3.531 | .730 | *F*(1,2135)=.227, | *F*(1,2135)=10.884, | *F*(1,2135)=.082, |
| ≥65 |  | 3.635 | .713 | 3.637 | .722 | *p*=.634 | *p*=.001 | *p*=.774 |
| Aesthetic Sens |  |  |  |  |  |  |  |  |
| <65 |  | 3.287 | .807 | 3.292 | .806 | *F*(1,2135)=2.349, | *F*(1,2135)=.157, | *F*(1,2135)=1.575, |
| ≥65 |  | 3.284 | .902 | 3.326 | .932 | *p*=.126 | *p*=.692 | *p*=.210 |
| Imagination |  |  |  |  |  |  |  |  |
| <65 |  | 3.481 | .775 | 3.488 | .779 | *F*(1,2135)=.714, | *F*(1,2135)=29.067, | *F*(1,2135)=.143, |
| ≥65 |  | 3.667 | .730 | 3.537 | .767 | *p*=.398 | *p*=.000 | *p*=.705 |
| Compassion |  |  |  |  |  |  |  |  |
| <65 |  | 3.686 | .769 | 3.680 | .772 | *F*(1,2135)=1.537, | *F*(1,2135)=108.980, | *F*(1,2135)=2.642, |
| ≥65 |  | 4.025 | .734 | 4.068 | .713 | *p*=.215 | *p*=.000 | *p*=.104 |
| Respectfulness |  |  |  |  |  |  |  |  |
| <65 |  | 3.897 | .776 | 3.874 | .791 | *F*(1,2135)=2.311, | *F*(1,2135)=110.416, | *F*(1,2135)=.009, |
| ≥65 |  | 4.263 | .647 | 4.242 | .656 | *p*=.129 | *p*=.000 | *p*=.926 |
| Trust |  |  |  |  |  |  |  |  |
| <65 |  | 3.244 | .747 | 3.260 | .743 | *F*(1,2135)=.001, | *F*(1,2135)=78.058, | *F*(1,2135)=1.309, |
| ≥65 |  | 3.561 | .700 | 3.545 | .734 | *p*=.970 | *p*=.000 | *p*=.253 |
| Organization |  |  |  |  |  |  |  |  |
| <65 |  | 3.823 | .842 | 3.789 | .844 | *F*(1,2135)=.906, | *F*(1, 2135)=55.885, | *F*(1,2135)=1.533, |
| ≥65 |  | 4.090 | .752 | 4.095 | .787 | *p*=.341 | *p*=.000 | *p*=.216 |
| Productiveness |  |  |  |  |  |  |  |  |
| <65 |  | 3.717 | .830 | 3.794 | .827 | *F*(1,2135)=6.142, | *F*(1,2135)=74.908, | *F*(1,2135)=1.115, |
| ≥65 |  | 4.013 | .694 | 4.075 | .714 | *p*=.013 | *p*=.000 | *p*=.291 |
| Responsibility |  |  |  |  |  |  |  |  |
| <65 |  | 3.801 | .791 | 3.787 | .793 | *F*(1,2135)=.003, | *F*(1,2135)=125.035, | *F*(1,2135)=1.091, |
| ≥65 |  | 4.178 | .611 | 4.193 | .630 | *p*=.957 | *p*=.000 | *p*=.296 |
| Responsibility ^a^ ^ |  |  |  |  |  |  |  |  |
| <65 |  | 3.861 | .775 | 3.860 | .788 | *F*(1,2063)=.127, | *F*(1,2063)=214.937, | *F*(1,2063)=.086, |
| ≥65 |  | 4.360 | .583 | 4.350 | .619 | *p*=.721 | *p*=.000 | *p*=.769 |
| Dutifulness ^a^ |  |  |  |  |  |  |  |  |
| <65 |  | 3.823 | .633 | 3.802 | .626 | *F*(1,2023)=18.996, | *F*(1,2023)=85.327, | *F*(1,2023)=7.417, |
| ≥65 |  | 4.112 | .482 | 4.023 | .496 | *p*=.000 | *p*=.000 | *p*=.007 |

*Note*. *N*=2,137. ^a^ Ns vary due to missing data. ^ From Roberts et al., 2005.
