# Supplementary material for: Change in five-factor model personality traits during the acute phase of the coronavirus pandemic
Source: PLoS One. 2020 Aug 6;15(8):e0237056. doi: 10.1371/journal.pone.0237056 (PMC7410194; doi:10.1371/journal.pone.0237056)
Supplement: S3 Table — N = 2,137. aNs vary due to missing data. ^ From Roberts et al. [14]. (DOCX) [file pone.0237056.s004.docx]

Table S3

*Interaction Between Time and Gender on Change in Personality Traits*

| Personality Trait |  | Pre |  | Post |  | Time | Gender | Time x Gender |
| --- | --- | --- | --- | --- | --- | --- | --- | --- |
|  |  | Mean | SD | Mean | SD |  |  |  |
| Neuroticism |  |  |  |  |  |  |  |  |
| Male |  | 2.456 | .706 | 2.427 | .737 | *F*(1,2135)=11.725, | *F*(1,2135)=93.676, | *F*(1,2135)=.318, |
| Female/other |  | 2.773 | .836 | 2.731 | .834 | *p*=.001 | *p*=.000 | *p*=.573 |
| Extraversion |  |  |  |  |  |  |  |  |
| Male |  | 3.167 | .605 | 3.120 | .609 | *F*(1,2135)=4.702, | *F*(1,2135)=16.137, | *F*(1,2135)=1.657, |
| Female/other |  | 3.071 | .673 | 3.078 | .675 | *p*=.030 | *p*=.000 | *p*=.198 |
| Openness |  |  |  |  |  |  |  |  |
| Male |  | 3.420 | .614 | 3.438 | .639 | *F*(1,2135)=1.503, | *F*(1,2135)=5.820, | *F*(1,2135)=.647, |
| Female/other |  | 3.489 | .633 | 3.493 | .639 | *p*=.220 | *p*=.016 | *p*=.421 |
| Agreeableness |  |  |  |  |  |  |  |  |
| Male |  | 3.614 | .624 | 3.625 | .635 | *F*(1,2135)=.102, | *F*(1,2135)=32.226, | *F*(1,2135)=2.392, |
| Female/other |  | 3.777 | .640 | 3.760 | .658 | *p*=.749 | *p*=.000 | *p*=.112 |
| Conscientiousness |  |  |  |  |  |  |  |  |
| Male |  | 3.841 | .683 | 3.860 | .697 | *F*(1,2135)=.008, | *F*(1,2135)=.340, | *F*(1,2135)=4.307, |
| Female/other |  | 3.878 | .726 | 3.857 | .751 | *p*=.927 | *p*=.560 | *p*=.038 |
|  |  |  |  |  |  |  |  |  |
| Anxiety |  |  |  |  |  |  |  |  |
| Male |  | 2.708 | .809 | 2.672 | .832 | *F*(1,2135)=11.790, | *F*(1,2135)=132.552, | *F*(1,2135)=.850, |
| Female/other |  | 3.125 | .930 | 3.063 | .925 | *p*=.001 | *p*=.000 | *p*=.357 |
| Depression |  |  |  |  |  |  |  |  |
| Male |  | 2.286 | .809 | 2.259 | .846 | *F*(1,2135)=6.980, | *F*(1,2135)=61.567, | *F*(1,2135)=.452, |
| Female/other |  | 2.580 | .962 | 2.535 | .967 | *p*=.008 | *p*=.000 | *p*=.501 |
| Emotional Vol |  |  |  |  |  |  |  |  |
| Male |  | 2.375 | .791 | 2.349 | .826 | *F*(1,2135)=2.516, | *F*(1,2135)=48.138, | *F*(2,2135)=.097, |
| Female/other |  | 2.613 | .934 | 2.596 | .920 | *p*=.113 | *p*=.000 | *p*=.756 |
| Sociability |  |  |  |  |  |  |  |  |
| Male |  | 2.978 | .860 | 2.962 | .861 | *F*(1,2135)=2.015, | *F*(1,2135)=8.739, | *F*(1,2135)=.021, |
| Female/other |  | 2.871 | .937 | 2.851 | .939 | *p*=.156 | *p*=.003 | *p*=.884 |
| Assertiveness |  |  |  |  |  |  |  |  |
| Male |  | 3.277 | .748 | 3.274 | .763 | *F*(1,2135)=8.530, | *F*(1,2135)=-0.722, | *F*(1,2135)=.809, |
| Female/other |  | 3.097 | .787 | 3.122 | .794 | *p*=.004 | *p*=.000 | *p*=.368 |
| Energy Level |  |  |  |  |  |  |  |  |
| Male |  | 3.294 | .681 | 3.349 | .694 | *F*(1,2135)=7.681, | *F*(2,2235)=5.180, | *F*(1,2235)=2.034, |
| Female/other |  | 3.244 | .836 | 3.262 | .823 | *p*=.006 | *p*=.023 | *p*=.154 |
| Curiosity |  |  |  |  |  |  |  |  |
| Male |  | 3.578 | .725 | 3.590 | .756 | *F*(1,2135)=.504, | *F*(1,2135)=4.729, | *F*(1,2135)=.104, |
| Female/other |  | 3.518 | .728 | 3.523 | .732 | *p*=.478 | *p*=.030 | *p*=.747 |
| Aesthetic Sens |  |  |  |  |  |  |  |  |
| Male |  | 3.140 | .852 | 3.170 | .879 | *F*(1,2135)=1.034, | *F*(1,2135)=72.945, | *F*(1,2135)=1.666, |
| Female/other |  | 3.440 | .780 | 3.436 | .772 | *p*=.309 | *p*=.000 | *p*=.197 |
| Imagination |  |  |  |  |  |  |  |  |
| Male |  | 3.543 | .735 | 3.553 | .765 | *F*(1,2135)=.572, | *F*(1,2135)=1.077, | *F*(1,2135)=.001, |
| Female/other |  | 3.511 | .801 | 3.520 | .796 | *p*=.449 | *p*=.300 | *p*=.971 |
| Compassion |  |  |  |  |  |  |  |  |
| Male |  | 3.647 | .752 | 3.670 | .762 | *F*(1,2135)=.215, | *F*(1,2135)=59.378, | *F*(1,2135)=1.692, |
| Female/other |  | 3.899 | .771 | 3.888 | .776 | *p*=.643 | *p*=.000 | *p*=.193 |
| Respectfulness |  |  |  |  |  |  |  |  |
| Male |  | 3.901 | .750 | 3.899 | .747 | *F*(1,2135)=3.410, | *F*(1,2135)=26.341, | *F*(1,2135)=2.774, |
| Female/other |  | 4.080 | .765 | 4.037 | .800 | *p*=.065 | *p*=.000 | *p*=.096 |
| Trust |  |  |  |  |  |  |  |  |
| Male |  | 3.295 | .717 | 3.306 | .731 | *F*(1,2135)=.377, | *F*(1,2135)=3.170, | *F*(1,2135)=.071, |
| Female/other |  | 3.352 | .779 | 3.356 | .770 | *p*=.539 | *p*=.075 | *p*=.790 |
| Organization |  |  |  |  |  |  |  |  |
| Male |  | 3.870 | .817 | 3.864 | .812 | *F*(1,2135)=3.408, | *F*(1,2135)=.440, | *F*(1,2135)=1.980, |
| Female/other |  | 3.910 | .841 | 3.867 | .869 | *p*=.065 | *p*=.507 | *p*=.160 |
| Productiveness |  |  |  |  |  |  |  |  |
| Male |  | 3.798 | .768 | 3.846 | .769 | *F*(1,2135)=4.941, | *F*(1,2135)=.785, | *F*(1,2135)=1.991, |
| Female/other |  | 3.788 | .849 | 3.778 | .856 | *p*=.026 | *p*=.376 | *p*=.158 |
| Responsibility |  |  |  |  |  |  |  |  |
| Male |  | 3.855 | .737 | 3.871 | .753 | *F*(1,2135)=.340, | *F*(1,2135)=3.467, | *F*(1,2135)=3.712, |
| Female/other |  | 3.936 | .796 | 3.906 | .798 | *p*=.560 | *p*=.063 | *p*=.054 |
| Responsibility ^a^ ^ |  |  |  |  |  |  |  |  |
| Male |  | 4.005 | .757 | 4.006 | .790 | *F*(1,2063)=.064, | *F*(1,2063)=1.648, | *F*(1,2063)=.129, |
| Female/other |  | 3.970 | .769 | 3.962 | .766 | *p*=.800 | *p*=.199 | *p*=.719 |
| Dutifulness ^a^ |  |  |  |  |  |  |  |  |
| Male |  | 3.896 | .601 | 3.865 | .560 | *F*(1,2023)=12.165, | *F*(1,2023)=.042, | *F*(1,2023)=.419, |
| Female/other |  | 3.898 | .621 | 3.852 | .608 | *p*=.000 | *p*=.838 | *p*=.517 |

*Note*. *N*=2,137. ^a^ Ns vary due to missing data. ^ From Roberts et al., 2005.
