# Supplementary material for: Change in five-factor model personality traits during the acute phase of the coronavirus pandemic
Source: PLoS One. 2020 Aug 6;15(8):e0237056. doi: 10.1371/journal.pone.0237056 (PMC7410194; doi:10.1371/journal.pone.0237056)
Supplement: S4 Table — Ns range from 2,105 to 2,109 due to missing data. Items are not given in full because the NEO-PI-3 is protected by copywrite. a Reported in the raw metric but reverse scored into the total Dutifulness score. (DOCX) [file pone.0237056.s005.docx]

Table S4

*Mean Change in Dutifulness Items between Pretest and Posttest*

| Personality Trait |  | Pretest | | Posttest | | Time | *p* | *η*^2^ |
| --- | --- | --- | --- | --- | --- | --- | --- | --- |
|  |  | Mean | SE | Mean | SE |  |  |  |
| Work/school when not feeling well |  | 3.70 | 1.11 | 3.16 | 1.30 | *F*(1,2104)=328.166 | .000 | .135 |
| Not dependable^a^ |  | 2.42 | 1.24 | 2.36 | 1.25 | *F*(1,2108)=4.891 | .027 | .002 |
| Pay debts |  | 4.07 | 1.15 | 4.06 | 1.17 | *F*(1,2108)=.129 | .720 | .000 |
| Ignore rules^a^ |  | 2.87 | 1.13 | 2.80 | 1.14 | *F*(1 2108)=6.421 | .011 | .003 |
| Can be counted on |  | 4.17 | .92 | 4.16 | .95 | *F*(1,2108)=.196 | .658 | .000 |
| Follow principles |  | 3.94 | .92 | 4.01 | .93 | F(1,2107)=11.780 | .001 | .006 |
| Do jobs carefully |  | 4.21 | .85 | 4.19 | .92 | F(1,2106)=1.131 | .288 | .001 |
| Perform tasks conscientiously |  | 4.19 | .882 | 4.22 | .88 | F(1,2107)=2.168 | .141 | .001 |

*Note*. *N*s range from 2,105 to 2,109 due to missing data. Items are not given in full because the NEO-PI-3 is protected by copywrite. ^a^ Reported in the raw metric but reverse scored into the total Dutifulness score.
